# SOX2 Expression Is Regulated by BRAF and Contributes to Poor Patient Prognosis in Colorectal Cancer

**DOI:** 10.1371/journal.pone.0101957

**Published:** 2014-07-10

**Authors:** Ida V. Lundberg, Anna Löfgren Burström, Sofia Edin, Vincy Eklöf, Åke Öberg, Roger Stenling, Richard Palmqvist, Maria L. Wikberg

**Affiliations:** 1 Department of Medical Biosciences, Pathology, Umeå University, Umeå, Sweden; 2 Department of Surgical and Perioperative Sciences, Surgery, Umeå University, Umeå, Sweden; West German Cancer Center, Germany

## Abstract

Sporadic colorectal cancer (CRC) is a common malignancy and also one of the main causes of cancer deaths worldwide. Aberrant expression of the transcription factor SOX2 has recently been observed in several cancer types, but its role in CRC has not been fully elucidated. Here we studied the expression of SOX2 in 441 CRC patients by immunohistochemistry and related the expression to clinicopathological and molecular variables and patient prognosis. SOX2 was expressed in 11% of the tumors and was significantly associated to *BRAF^V600E^* mutation, but not to *KRAS* mutations (codon 12 and 13). SOX2 positivity was correlated to poor patient survival, especially in *BRAF^V600E^* mutated cases. *In vitro* studies showed that cells expressing the constitutively active *BRAF^V600E^* had increased SOX2 expression, a finding not found in cells expressing *KRAS^G12V^*. Furthermore, blocking downstream BRAF signalling using a MEK-inhibitor resulted in a decreased expression of SOX2. Since SOX2 overexpression has been correlated to increased migration and invasion, we investigated the SOX2 expression in human CRC liver metastasis and found that a SOX2 positive primary CRC also had SOX2 expression in corresponding liver metastases. Finally we found that cells overexpressing SOX2 *in vitro* showed enhanced expression of FGFR1, which has been reported to correlate with liver metastasis in CRC. Our novel findings suggest that SOX2 expression is partly regulated by BRAF signalling, and an increased SOX2 expression may promote CRC metastasis and mediate a poor patient prognosis.

## Introduction

Sporadic colorectal cancer (CRC) is a common malignancy in the western world and one of the main causes of cancer deaths. The high mortality rate, due to occult or clinically identified disseminated disease already at diagnosis, emphasizes the importance of a higher understanding of the biological events leading to an invasive cancer. This knowledge is important to predict patient prognosis and create new, powerful therapies. The adenoma to carcinoma sequence depicture the genetic events needed for a normal colon epithelia to be transformed into a malignant phenotype in most of the sporadic CRC cases [1]. Since the metastatic process in CRC is not as fully understood, it is hard to elucidate why some tumors become more aggressive and metastasize more easily than others. Identification of molecular markers expressed in invasive tumors that can predict poor patient prognosis is therefore an important research subject.

SRY (sex determining region Y)-box 2 (SOX2) is a member of the large *SOX* gene family, comprising transcription factors known to be important in the regulation of developmental processes and cell type specification [2]. The key member SOX2 plays essential roles in the maintenance of cell pluripotency and self-renewal in both embryonic stem cells [3] and in induced pluripotent stem cells [4]. Recently it has also been reported that self-renewal of cancer stem cells is maintained by SOX2 [5], suggesting an ongogenic role of SOX2. Overexpression of SOX2 can be seen in CRC [6]–[8] as well as in several other malignancies such as breast, pancreatic and gastric cancers [9]–[11], demonstrating its involvement in carcinogenesis. In addition, SOX2 has been suggested to be involved in CRC cell migration, invasion and metastasis, where matrix metalloproteinase 2 (MMP2) has been proposed as a potential mediator for the SOX2 effect [6], but the exact mechanisms still need to be discovered.

In the present study we evaluated SOX2 expression in primary CRC, as well as in samples of corresponding liver metastasis, and correlated our findings to patient prognosis and molecular tumor characteristics. Our results suggest that SOX2 expression is, at least partly, regulated by BRAF, and that expression of BRAF^V600E^ in a stage dependent manner correlates to a poor patient prognosis.

## Materials and Methods

### Ethics Statement

In the present study, the handling of tissue samples and patient data was approved by the research ethical committee at Umeå University Hospital (Regional Ethical Review Board in Umeå, Sweden). This includes the procedure whereby patients verbally gave their informed consent, which was documented in each patient record and considered by the Ethics Committee to be sufficient. Each tissue sample was registered as a case number and year in the database used for the analyses, and names or personal identification were not indicated.

### Clinical samples

The CRC tissue samples included in the study were from the Colorectal Cancer in Umeå Study (CRUMS), which consists of patients that have been surgically resected for primary CRC between 1995 and 2003 at Umeå University Hospital, Sweden. Histopathological classifications of all cases were performed by one pathologist by reviewing routinely stained tumor sections. Clinical data were obtained by reviewing the patient records, and survival data were collected during autumn 2012.

13 patients with archival tissue from both a primary colorectal adenocarcinoma and corresponding distant liver metastasis that were diagnosed in the same time interval as CRUMS were included in the present study. These were identified using the computerized patient record database at the Department of Clinical Pathology, Umeå University Hospital, Sweden. The tumors were graded and diagnosed by pathologists at the time of surgery or biopsy.

### Immunohistochemistry

CRC specimens were formalin-fixed and paraffin embedded according to routine protocols at the Department of Clinical Pathology, Umeå university Hospital, Sweden. They were cut at 4-µm and then dried, deparaffinized and rehydrated. Anti-SOX2 polyclonal antibody [12]–[14] (Abcam, Cambridge, UK) was used at a concentration of 1∶500 in a semiautomatic staining machine (Bench Mark Ultra, Ventana Inc) and visualized by iVIEW DAB Detection kit (Ventana Inc)). The slides were counterstained with hematoxylin.

For CRUMS, 449 cases were immunohistochemically stained, but due to lack of tumor material (n = 7) or repeated tissue loss during antigen retrieval step (n = 1), eight of them could not be analyzed for SOX2 staining. All the 13 patients with corresponding metastasis were successfully stained, and all could be analyzed for SOX2-positive cells. The specimens were reviewed under light microscopy, and each sample was evaluated two times by the same observer and in cases with discrepant scoring, a third final evaluation was made. Nuclear staining was assessed as negative or positive. Occasional cytoplasmic or stromal staining was not analyzed.

### Statistical analyses

Associations between SOX2 expression and different clinicopathological variables were analyzed using Pearson's χ^2^ tests. To estimate cancer-specific survival, Kaplan-Meier survival analysis was used, and comparisons between groups were made using the log-rank test. Patients in the CRUMS cohort who died within one month from surgery due to postoperative complications (n = 37) were excluded from the survival analyses. Cox proportional hazard models were used for multivariate analyses. Cancer-specific events were defined as death with known disseminated or recurrent disease, and cases were censored at the end of follow-up or at time of death by other causes. SPSS/PASW statistical software version 20 (SPSS Inc., Chicago, Illinois, USA) was used for the statistical analyses. Gene expression levels were compared using two tailed Student's *t* test. Each bar represents an average of three independent experiments and the error bars illustrate the standard deviation. *p*<0.05 was considered statistically significant for all analyses.

### Cell lines and cell culture

In the present study, the colon cancer cell line Caco2 (American Type Culture Collection, Manassas, VA, USA) was grown in Dulbecco's modified Eagle's medium (DMEM) with glutamax supplemented with 10% fetal bovine serum (FBS) (Gibco, Life Technologies, Stockholm, Sweden) and maintained at 37°C and 5% CO_2_. Generation of the stable transfectants expressing SOX2 (Caco2-SOX2), mutant BRAF (Caco2-BRAF^V600E^) or mutant KRAS (Caco2-KRAS^G12V^) was performed by transfecting Caco2-cells with pcDNA3.3-SOX2 (Derrick Rossi, Childrens Hospital Boston, USA, via Addgene), pMCEF-BRAFV^600E^ (kindly provided by Prof R. Marais) or pcDNA3-KRAS^G12V^ (kind gift from Dr N. Ignatenko) using Caco-2 Transfection Reagent (Altogen Biosystems, Las Vegas, NV, USA) according to the manufacturer's instructions. Between 48 and 72 hours after exposure to the DNA, transfected cells were selected with 800 µg/ml G418 (Gibco, Life Technologies, Stockholm, Sweden). Medium containing G418 was changed twice a week.

To block BRAF signaling in Caco2 and Caco2-BRAF^V600E^ cells, 20 µM MEK inhibitor PD98059 (Santa Cruz Biotechnology, Santa Cruz, CA, USA), or DMSO as control, was added to the cells following incubation for 24 or 48 h.

### RT-PCR

Total RNA was isolated from cells using the NucleoSpin RNA II kit (Macherey-Nagel, Duren, Germany), and cDNA was synthesized with the SuperScript II Reverse Transcriptase (Invitrogen, Life Technologies, Stockholm, Sweden) according to manufacturer's protocols. Primers used in the study were from DNA Technology A/S (Aarhus, Denmark) and their sequences were as follows: GAPDH forward: 5′-TGCACCACCAACTGCTTAGC-3′, reverse: 5′-GGCATGGACTGTGGTCATGAG-3′. SOX2 forward: 5′-AACCCCAAGATGCACAACTC-3′, reverse: 5′-CGGGGCCGGTATTTATAATC-3′. FGFR1 forward: 5′-AGGCTACAAGGTCCGTTATGC-3′, reverse: 5′- TGCCGTACTCATTCTCCACAA-3′. FGFR2 forward: 5′-TTAAGCAGGAGCATCGCATTG-3′, reverse: 5′- GGGACCACACTTTCCATAATGAG-3′. FGFR3 forward: 5′-CCTCGGGAGATGACGAAGAC-3′, reverse: 5′-CGGGCCGTGTCCAGTAAGG-3′. FGFR4 forward: 5′-TGCAGAATCTCACCTTGATTACA-3′, reverse: 5′-GGGGTAACTGTGCCTATTCG-3′. Every PCR-reaction contained 25 ng cDNA and each sample was run in duplicates. The experiments were repeated three times. Standard deviations were calculated of the mean of triplicate reactions. The RT-PCR-reactions were performed on Taqman 7900HT (Applied Biosystems, Life Technologies, Stockholm, Sweden) and following cycling parameters were used: 50°C for 2 min and then an initial denaturation at 95°C for 10 min, followed by 40 cycles of 95°C for 15 s and 60°C for 60 s. Gene expressions were normalized to GAPDH.

### Western blot

To analyze SOX2 expression in the stable Caco2-SOX2 transfectant, cells were lysed in lysis buffer (100 mM NaCl, 50 mM Tris pH 7.5, 1% Triton X-100, 1 mM EDTA pH 8.0, 15 mM MgCl_2_, protein inhibitors) before proteins were separated by SDS PAGE and transferred to a PVDF membrane (GE Healthcare, Uppsala, Sweden). The blot was incubated with primary SOX2 antibody (1∶1000, Cell Signaling Technology, Danvers, MA, USA) and secondary antibody conjugated to horseradish peroxidase (GE Healthcare, Uppsala, Sweden) according to manufacturer's instructions. The blot was developed with ECL Select Western Blotting Detection Reagent (GE Healthcare, Uppsala, Sweden).

### Digital droplet PCR

Genomic DNA was isolated from the cells using the Nucleospin Tissue kit (Macherey-Nagel, Duren, Germany) according to manufacturer's instructions.

Digital droplet PCR (ddPCR, Bio-Rad Laboratories, Hercules, CA, USA) was used to verify the Caco2 transfectants expressing mutant *BRAF^V600E^* (Caco2-BRAF^V600E^) and *KRAS^G12V^* (Caco2-KRAS^G12V^). The ddPCR-method has been presented thoroughly elsewhere [15], [16]. Briefly, the ddPCR allows detection and quantification of both mutation and wild type in the same reaction using the fluorophores FAM and HEX conjugated to sequence specific probes. In the ddPCR-method, a PCR sample of 20 µl is partitioned into 20 000 nanoliter droplets giving about 20 000 reads.

To verify a successful transfection of Caco2-BRAF^V600E^, the primers and probes were as follows: forward: 5′-GCACAGGGCATGGATTACTTACA-3′, reverse: 5′-ATCCAGACAACTGTTCAAACTGATG-3′, wild type probe: 5′-56-FAM/TTGGTCTAGCTACAGTGAAAT/3BHQ_1-3′, mutation probe: 5′-5HEX/TTGGTCTAGCTACAGAGAAAT/3BHQ_1-3′ (DNA Technology A/S, DNA Technology A/S) [17], [18]. The PCR was performed in a T100 Thermal Cycler(Bio-Rad Laboratories, Hercules, CA, USA) using the program: 95°C for 10 min; 40x cycles of 95°C for 15 s and 56°C for 1 min (ramp rate 2°C/sec); and 98°C for 10 min. 900 nM of the primers, and 250 nM of respective probe was used.

For detection of successful transfection of Caco2-KRAS^G12V^, assays for ddPCR were used (PrimePCR ddPCR Mutation Assay: KRAS p.G12V assay, Human; PrimePCR ddPCR Mutation Assay: KRAS wild type for p.G12V assay, Human, Bio-Rad Laboratories, Hercules, CA, USA) with PCR-conditions according to manual provided by the company: 95°C for 10 min; 40x cycles of 94°C for 30 s and 55°C for 1 min (ramp rate 2°C/sec); and 98°C for 10 min.

Every PCR reaction contained 50 ng DNA and the droplets were prepared in a QX100 droplet generator (Bio-Rad Laboratories, Hercules, CA, USA). The final PCR-product was detected in a QX200 droplet reader (Bio-Rad Laboratories, Hercules, CA, USA) and the result analyzed with QuantaSoft software, Version 1.4 (Bio-Rad Laboratories, Hercules, CA, USA).

### CpG island methylator phenotype (CIMP) status

Tumor CIMP status was determined by the MethyLight method with primer and probe sequences that are previously described [19], [20]. For the eight genes in the CIMP panel (*CDKN2A*, *MLH1*, *CACNA1G*, *NEUROG1*, *RUNX3*, *SOCS1*, *IGF2* and *CRABP1*) [20] the percent of methylated reference (PMR) was calculated, where PMR >10 was considered as positive [19]. Tumors were classified as CIMP negative (no promoter hypermethylation), CIMP low (one to five genes methylated) or CIMP high (six to eight genes methylated) [20].

### Microsatellite instability (MSI) screening status

Mismatch repair proteins were analyzed by immunohistochemistry as previously described [20]. Briefly, formalin-fixed and paraffin embedded CRC tissue was examined for expression of four mismatch repair proteins (MLH1, MSH2, MSH6 and PMS2). A sample considered having a positive MSI screening status lacked nuclear staining in tumor cells for at least one of the proteins and is referred to as MSI. A negative MSI screening status had expression of all four genes and was referred to as microsatellite stable (MSS).

### BRAF^V600E^ mutational status

The Taqman allelic discrimination assay, described in detail elsewhere [17], was used for detection of the *BRAF^V600E^* mutation (reagents from Applied Biosystems, Life Technologies, Stockholm, Sweden).

### KRAS sequencing

Mutational analysis of *KRAS* has been explained elsewhere [21]. The sequencing was carried out using Big Dye v. 3.1 (Applied Biosystems, Life Technologies, Stockholm, Sweden) and primers used were: forward: 5′-TGTAAAACGACGGCCAGTGAGTTTGTATTAAAAGGTACTGG-3′ and reverse: 5′-CAGGAAACAGCTATGACCTCTGTATCAAAGAATGGTCCT-3′.

## Results

### SOX2 expression in CRC correlates with tumor grade, TNM stage and *BRAF* mutation

**Figure 1:**
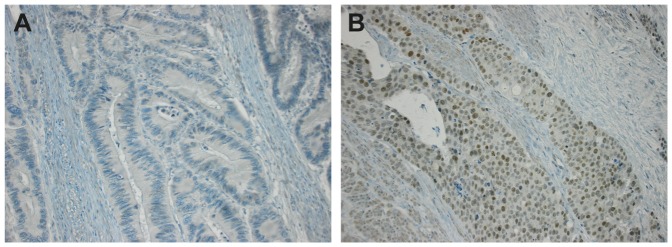
Representative immunohistochemical stainings of SOX2 expression in CRC tissue. (A) Negative nuclear SOX2 staining in a moderately differentiated CRC. (B) Positive nuclear SOX2 staining in a poorly differentiated CRC.

Nuclear SOX2 expression in tumor cells was evaluated in 441 CRC patient samples by immunohistochemistry, where the expression was assessed as either positive or negative (Figure 1). In the positive cases, SOX2 expression was never seen in the whole tumor, but positive nuclei were found in limited parts. Occasional stromal or cytoplasmic staining was not evaluated. 47 (10.7%) of the CRC samples displayed tumor cells expressing SOX2 and the expression in relation to different clinicopathological characteristics is shown in Table 1. In our patient cohort, SOX2 expression was found to be significantly associated with a high tumor grade (*p* = 0.004) and TNM stage (*p* = 0.034). SOX2 expression was also highly correlated to *BRAF* mutation (*p*<0.001), but surprisingly no correlation to *KRAS* mutations could be seen (*p* = 0.928).

**Table 1:** SOX2 expression in relation to clinicopathological characteristics in CRC.

	SOX2 negative	SOX2 positive	*p* value^a^
**Frequencies, n (%)**	394 (89.3)	47 (10.7)	
**Sex, n (%)**			0.032
Male	224 (92.2)	19 (7.8)	
Female	170 (85.9)	28 (14.1)	
**Age, n (%)**			0.548
≤59 years	70 (86.4)	11 (13.6)	
60–69 years	97 (90.7)	10 (9.3)	
70–79 years	147 (91.3)	14 (8.7)	
≥80 years	80 (87.0)	12 (13.0)	
**TNM stage, n (%)** b			0.034
I	62 (93.9)	4 (6.1)	
II	153 (92.2)	13 (7.8)	
III	81 (89.0)	10 (11.0)	
IV	87 (82.1)	19 (17.9)	
**Localization, n (%)** b			0.283
Right colon	122 (85.9)	20 (14.1)	
Left colon	118 (90.1)	13 (9.9)	
Rectum	149 (91.4)	14 (8.6)	
**Grade, n (%)** b			0.004
Highly to moderately differentiated	202 (93.5)	14 (6.5)	
Moderately to poorly differentiated	185 (84.9)	33 (15.1)	
**MSI screening status, n (%)** b			0.725
MSI	60 (88.2)	8 (11.8)	
MSS	321 (89.7)	37 (10.3)	
**CIMP status, n (%)** b			0.193
CIMP-negative^c^	198 (91.7)	18 (8.3)	
CIMP-low^c^	145 (87.9)	20 (12.1)	
CIMP-high^c^	47 (83.9)	9 (16.1)	
***BRAF^V600E^*** **, n (%)** b			<0.001
wild type	339 (91.4)	32 (8.6)	
mutated	47 (75.8)	15 (24.2)	
***KRAS*** ** (codon 12, 13), n (%)** b			0.928
wild type	315 (89.2)	38 (10.8)	
mutated	72 (88.9)	9 (11.1)	

Abbreviations: MSI, microsatellite instability; MSS, microsatellite stable; CIMP, CpG island methylator phenotype (according to an eight-gene CIMP panel).

a χ2 test.

b The following numbers of missing cases were present: TNM stage, 12; localization, 5; grade, 7; MSI screening status, 15; CIMP status, 4; BRAF V600E, 8; KRAS, 7.

c CIMP negative, no promoter hypermethylation; CIMP low, one to five genes methylated; CIMP high, six to eight genes methylated.

### SOX2 expression is correlated to a poor patient survival

**Figure 2:**
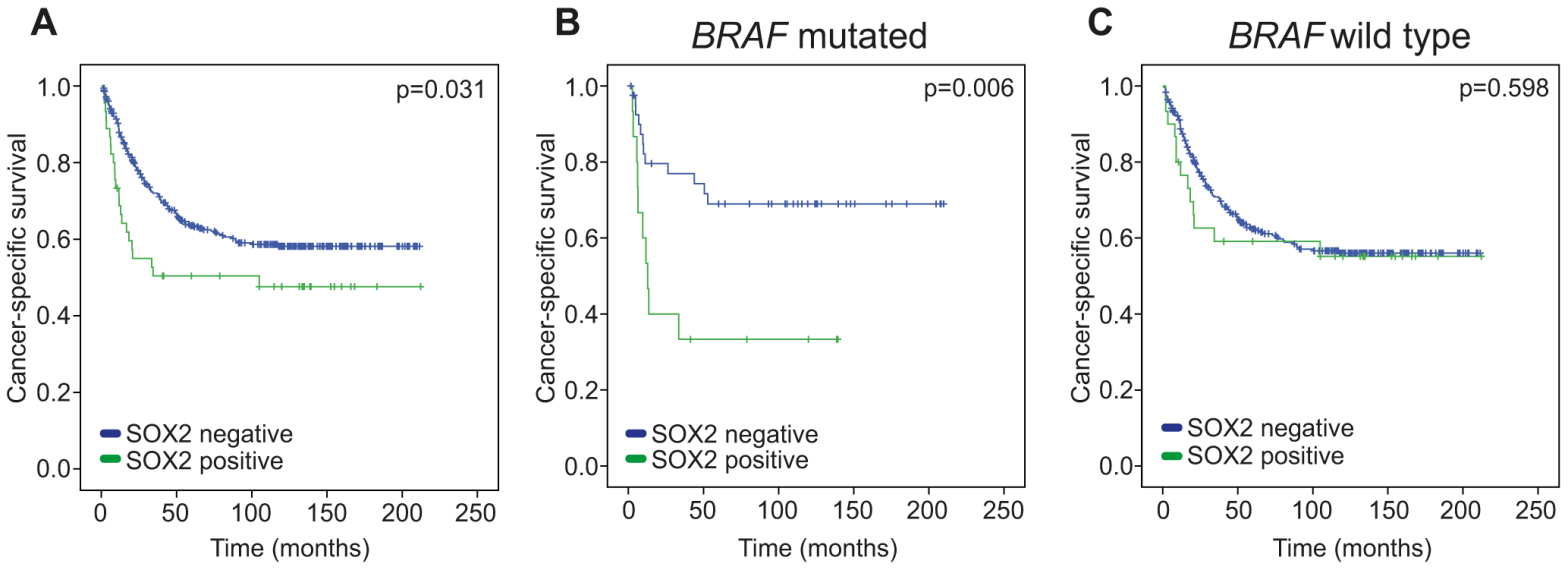
Cancer-specific survival analysis according to SOX2 expression. Shown are Kaplan-Meier plots of cancer-specific survival in (A) all CRC patients, (B) *BRAF^V600E^* mutated CRC patients or (C) *BRAF* wild type CRC patients.

**Figure 3:**
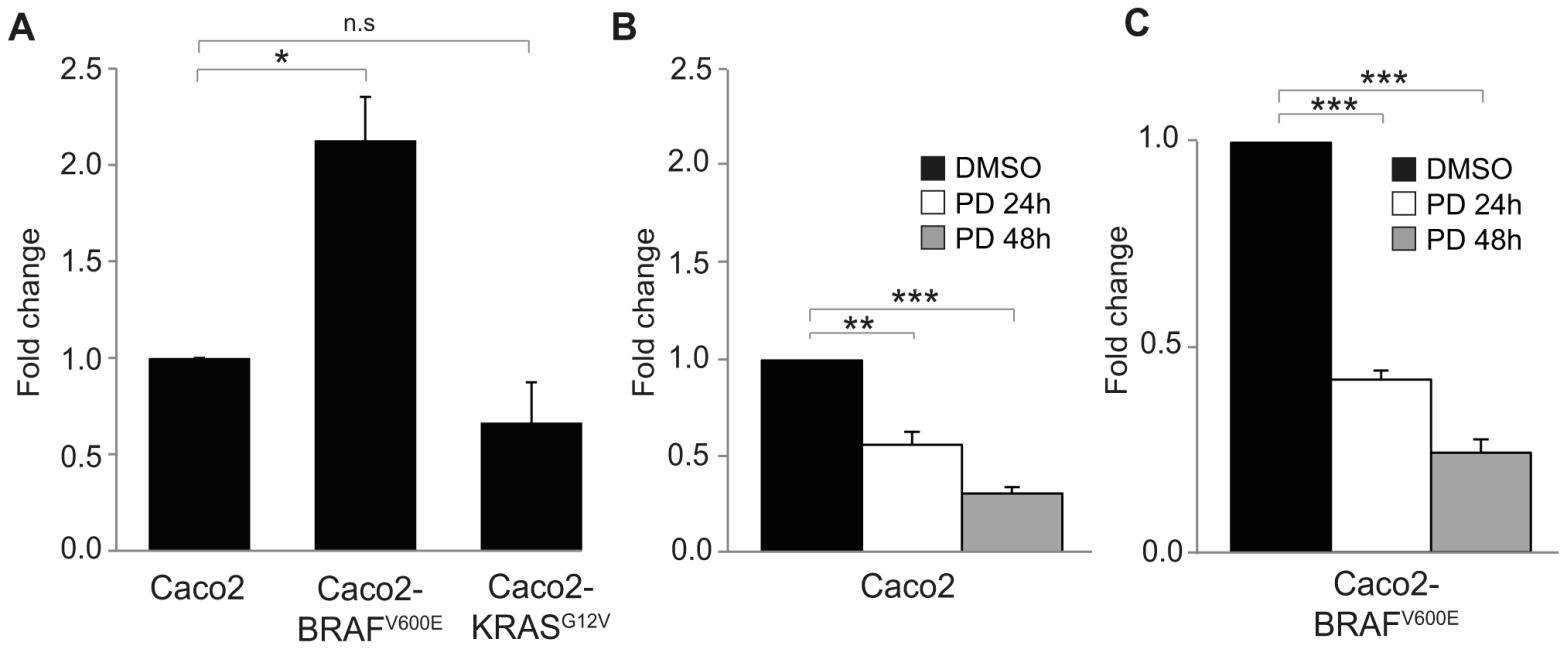
Real Time PCR analyses of SOX2 expression in CRC cell lines. (A) Caco2 cells, Caco2 cells stably expressing *BRAF* mutation (Caco2-BRAF^V600E^) and Caco2 cells stably expressing *KRAS* mutation (Caco2-KRAS^G12V^). SOX2 expression in Caco2 was set as 1. (B) Caco2 after cultivation with MEK-inhibitor, 24 or 48 h, or DMSO for 48 h as control. SOX2 expression in Caco2 treated with DMSO was set as 1. (C) Caco2-BRAF^V600E^ after cultivation with MEK-inhibitor, 24 or 48 h, or DMSO for 48 h as control. SOX2 expression in Caco2-BRAF^V600E^ treated with DMSO was set as 1. PD: PD98059 (MEK-inhibitor), *p<0.05, **p<0.01, ***p<0.001, n.s: non-significant *p*-value.

Cancer-specific survival analyses revealed that patients with SOX2 positive tumors had a poorer prognosis than patients with SOX2 negative tumors (Figure 2a). This association was even stronger when only the *BRAF* mutated tumors was analyzed (Figure 2b), whilst no difference of SOX2 expression on survival was seen in *BRAF* wild type tumors (Figure 3c). SOX2 expression did not have any effect on patient prognosis in *KRAS* mutated tumors (*p* = 0.676). In a multivariate Cox proportional hazard model including age, sex, *BRAF* mutation, and SOX2 expression, the poor prognosis for patients with SOX2 expression versus no SOX2 expression retained statistical significance (hazard ratio (HR) = 1.64, 95% CI 1.04–2.58, *p* = 0.032). When further adjusting for stage in the multivariate analysis, the prognostic impact of SOX2 expression was lost (HR = 1.04, 95% CI 0.65–1.67, *p* = 0.878), emphasizing stage dependency.

### SOX2 expression is regulated by BRAF *in vitro*

As SOX2 expression correlated with mutated *BRAF* in our patient cohort, we continued to analyze their molecular relation *in vitro*. The CRC cell line Caco2, with endogenous BRAF and KRAS wild type, was transfected with either BRAF^V600E^ or KRAS^G12V^ in order to establish cell lines stably expressing mutant *BRAF* (Caco2-BRAF^V600E^, Figure S1a) or mutant *KRAS* (Caco2-KRAS^G12V^, Figure S1b). By RT-PCR analyses we found that Caco2-BRAF^V600E^ expressed about twice as high SOX2-levels compared to Caco2 (*p* = 0.013), an increase which was not seen in Caco2-KRAS^G12V^ (Figure 3a). These results, in concordance with our data from the patient cohort (Table 1), suggest that the expression of SOX2 is regulated by mutated BRAF. The *BRAF^V600E^* mutation renders BRAF in a constitutively active state, stimulating the MEK/ERK signaling cascade in the absence of extracellular stimuli [22]. Indeed, blocking BRAF downstream signaling in Caco2-BRAF^V600E^ cells using the MEK-inhibitor PD98059, resulted in a decreased expression of SOX2 (Figure 3c), suggesting that it is mainly the well characterized MEK activating activity of BRAF that regulates SOX2. Furthermore, the low endogenous levels of SOX2 in Caco2 cells were also decreased by the MEK-inhibitor (Figure 3b), implying that BRAF responding to endogenous activation signals also regulates SOX2 expression. Together these results support the role of BRAF as an upstream regulator of SOX2 expression.

### Primary SOX2 positive CRC has SOX2 positive corresponding liver metastasis

Given that fact that SOX2 expression is correlated to a worse patient prognosis, we wanted to study SOX2 expression in primary tumors as well as corresponding distant metastasis. For this, 13 patients with archival tissue from both a primary colorectal adenocarcinoma and distant liver metastasis were included in the study and nuclear SOX2 expression was evaluated in epithelial cells. Two of the 13 primary tumors were SOX2 positive, whereas the other eleven tumors were SOX2 negative. Interestingly, SOX2 positive primary tumors were also SOX2 positive in corresponding liver metastasis, while SOX2 negative tumors had SOX2 negative liver metastasis (Table 2). Noteworthy, these SOX2 positive metastasis were morphologically more alike the tumor compartments having SOX2 positive nuclei than the rest of the primary tumor (data not shown).

**Table 2:** SOX2 expression in primary CRC and corresponding liver metastasis.

	CRC primary tumors	
Liver metastases	SOX2 negative	SOX2 positive
**SOX2 negative**	11	0
**SOX2 positive**	0	2

### SOX2 enhance expression of FGFR1

Deregulation of fibroblast growth factor receptors (FGFRs) is seen in CRC as well as in other cancers [23], [24]. Since it has been suggested that SOX2 regulate the expression of FGFR3 [7], we wanted to study if SOX2 altered the expression of the FGFRs in our *in vitro* system.

**Figure 4:**
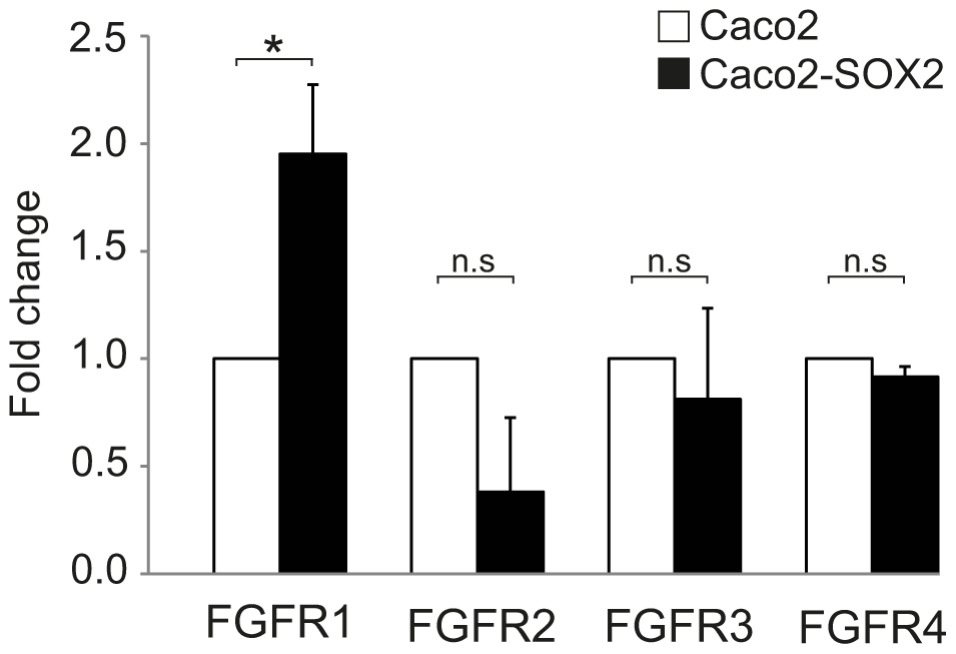
Expression of FGFR1 is increased in Caco2-SOX2 compared to Caco2. Expression of FGFR1, FGFR2, FGFR3 and FGFR4 by RT-PCR analysis in Caco2 cells and Caco2 cells stably overexpressing SOX2 (Caco2-SOX2). The expression in Caco2 was set as 1. *p<0.05, n.s: non-significant *p*-value.

A cell line overexpressing SOX2 was established by transfecting the CRC cell line Caco2 with SOX2 (Caco2-SOX2, Figure S2). The expression of FGFR1-4 was analyzed by RT-PCR in Caco2 cells and Caco2-SOX2 cells. FGFR1 expression was found to be twice as high in Caco2-SOX2 as in Caco2 (*p* = 0.036), while no significant differences were seen regarding FGFR2, FGFR3 or FGFR4 (Figure 4).

## Discussion

In this study we investigated SOX2 expression in CRC in correlation to several clinicopathological and molecular variables and its effect on patient prognosis. 11% of the tumors were SOX2 positive, and the expression correlated to tumor grade, TNM stage and *BRAF* mutation. Additionally, we found that SOX2 expression predicted a poorer patient prognosis in a stage dependent manner, in particular in *BRAF* mutated cases. Finally, we introduce SOX2 as possible regulator of FGFR1 expression.

Because of the asserted involvement of SOX2 in CRC tumorigenesis [25] we wanted to study the expression of SOX2 in our patient cohort CRUMS. We found that 11% of the tumors expressed SOX2 and by comparing the expression to different clinicopathological characteristics we could see that SOX2 expression was correlated to poorly differentiated tumors (high grade). This fits with the knowledge that SOX2 is a known stem cell marker, and has been suggested to be expressed in cancer stem cells [5], [26]. As SOX2 often were expressed in a limited part of the tumor, we speculate that these cells might be representing the cancer stem cell niche in these particular tumors. We further found that SOX2 expression was correlated to poor patient prognosis in a stage dependent manner, which is consistent with some previous studies [6], [27], [28].

SOX2 has been described by others to enhance the migratory and invasive effect of CRC cells [6], [7] as wells as of other cancer cell types [29]–[31], which implies that SOX2 expressing cells might harbor a higher metastatic capacity. In CRC it has also been suggested that the expression of SOX2 can predict tumor metastasis [6]. However, no studies exist today of SOX2 expression in tumor metastases. Here we have shown that corresponding liver metastases to SOX2 positive primary tumors also harbor SOX2 positive tumor cells. Although these patient tissue specimens were very few, it still indicates that SOX2 positive cells are more likely to migrate. It would of course be both interesting and necessary to study this in a larger patient material for verification. The fact that the SOX2 positive cells only made up a small proportion of the whole tumor, suggests that these cells may display a more invasive phenotype than the surrounding tumor cells. Another supporting evidence for a more invasive behavior of SOX2 positive tumor cells is that the metastases were morphologically more similar to the parts of the tumor with SOX2 positive nuclei. Even though the morphological comparison between primary tumors and metastases could only be studied in two cases, we find it likely that it may reflect specific cells that actually give rise to distant metastases.

SOX2 expression was correlated to *BRAF* mutation in our tissue cohort, but no correlation could be seen between SOX2 expression and *KRAS* mutations. Our *in vitro* finding that an increased SOX2 expression was seen in cells expressing BRAF^V600E^ mutation but not KRAS^G12V^ mutation, confirms this correlation. The RAS/RAF/MAP kinase cascade is a pathway that is implicated in many important cellular functions such as cell growth, division and differentiation [32], and its included proteins are frequently mutated in CRC. Of all CRCs, 30–40% are mutated in the *KRAS* gene and 5–15% in the *BRAF* gene [21], [33]. These two mutations are believed to be mutually exclusive in CRC [21], [34], and they are both associated with poor patient prognosis [21], [35]–[37]. Although they are involved in the same pathway, we can see that *KRAS* and *BRAF* mutated CRCs have different morphological appearances. It is interesting to speculate that the association of *BRAF* mutation with SOX2 expression might explain part of that morphological difference. We continued to analyze the correlation of BRAF and SOX2 expression in our *in vitro* cell culture system. Indeed, SOX2 expression was found to be upregulated in cells expressing the constitutively active BRAF^V600E^. Furthermore, by blocking BRAF downstream signaling, endogenous SOX2 as well as SOX2 expression induced by BRAF^V600E^ was decreased, demonstrating that SOX2 expression is at least partly regulated by the well characterized BRAF/MEK pathway. To our knowledge, this is the first study showing that SOX2 expression is regulated by BRAF signaling. Another interesting finding was that the negative prognostic effect of SOX2 expression was restricted to *BRAF* mutated patients, which indicates that it is SOX2 expression in combination with *BRAF* mutation that mostly contributes to the poor prognosis.

It is well known that aberrant expression of fibroblast growth factor receptors (FGFRs) can drive tumor progression [23], [24]. The FGFR family is comprised of four genes, and it has been shown that at least the *FGFR3* gene is regulated by SOX2 in CRC [7]. Our cell line overexpressing SOX2, Caco2-SOX2, had twice as high FGFR1 expression as Caco2 cells, implying that FGFR1 expression might be regulated by SOX2. Others have shown that overexpression of FGFR1 is found in CRC [38] and that it is correlated to liver metastasis [39]. A recent study has also suggested that elevated expression of both SOX2 and FGFR1 is correlated to poor prognosis in small cell lung cancer [40]. Together, these findings suggest that SOX2 in part through upregulation of FGFR1 might enhance distant spreading of tumor cells to the liver, thereby causing a poorer patient survival. However, additional studies are needed to reveal the role and mechanism of SOX2 and FGFR1 in CRC.

In conclusion, this study shows that SOX2 expression is correlated to a poor prognosis in CRC patients and it identifies for the first time that SOX2 expression partly is regulated by BRAF. These findings in combination with the observed correlation between SOX2 positivity in both primary tumor and corresponding metastasis, suggest that SOX2 positive tumor cells have an enhanced capacity to metastasize.

## Supporting Information

Figure S1 **Successful transfection of pMCEF-BRAF^V600E^ or pcDNA3-KRAS^G12V^ into Caco2 colon cancer cell line.**
(PDF)

Figure S2 **Caco2-SOX2 cells have an increased SOX2 expression at both mRNA and protein level.**
(PDF)

## References

[1] Fearon ER, Vogelstein B (1990) A genetic model for colorectal tumorigenesis. Cell 61: 759–767. 2188735 10.1016/0092-8674(90)90186-i

[2] Sarkar A, Hochedlinger K (2013) The sox family of transcription factors: versatile regulators of stem and progenitor cell fate. Cell Stem Cell 12: 15–30. 23290134 10.1016/j.stem.2012.12.007 PMC3608206

[3] Avilion AA, Nicolis SK, Pevny LH, Perez L, Vivian N, et al (2003) Multipotent cell lineages in early mouse development depend on SOX2 function. Genes Dev 17: 126–140. 12514105 10.1101/gad.224503 PMC195970

[4] Takahashi K, Yamanaka S (2006) Induction of pluripotent stem cells from mouse embryonic and adult fibroblast cultures by defined factors. Cell 126: 663–676. 16904174 10.1016/j.cell.2006.07.024

[5] Basu-Roy U, Seo E, Ramanathapuram L, Rapp TB, Perry JA, et al (2012) Sox2 maintains self renewal of tumor-initiating cells in osteosarcomas. Oncogene 31: 2270–2282. 21927024 10.1038/onc.2011.405 PMC3243769

[6] Han X, Fang X, Lou X, Hua D, Ding W, et al (2012) Silencing SOX2 induced mesenchymal-epithelial transition and its expression predicts liver and lymph node metastasis of CRC patients. PLoS One 7: e41335. 22912670 10.1371/journal.pone.0041335 PMC3422347

[7] Fang X, Yu W, Li L, Shao J, Zhao N, et al (2010) ChIP-seq and functional analysis of the SOX2 gene in colorectal cancers. OMICS 14: 369–384. 20726797 10.1089/omi.2010.0053

[8] Park ET, Gum JR, Kakar S, Kwon SW, Deng G, et al (2008) Aberrant expression of SOX2 upregulates MUC5AC gastric foveolar mucin in mucinous cancers of the colorectum and related lesions. Int J Cancer 122: 1253–1260. 18027866 10.1002/ijc.23225

[9] Rodriguez-Pinilla SM, Sarrio D, Moreno-Bueno G, Rodriguez-Gil Y, Martinez MA, et al (2007) Sox2: a possible driver of the basal-like phenotype in sporadic breast cancer. Mod Pathol 20: 474–481. 17334350 10.1038/modpathol.3800760

[10] Sanada Y, Yoshida K, Ohara M, Oeda M, Konishi K, et al (2006) Histopathologic evaluation of stepwise progression of pancreatic carcinoma with immunohistochemical analysis of gastric epithelial transcription factor SOX2: comparison of expression patterns between invasive components and cancerous or nonneoplastic intraductal components. Pancreas 32: 164–170. 16552336 10.1097/01.mpa.0000202947.80117.a0

[11] Li XL, Eishi Y, Bai YQ, Sakai H, Akiyama Y, et al (2004) Expression of the SRY-related HMG box protein SOX2 in human gastric carcinoma. Int J Oncol 24: 257–263. 14719100

[12] Ruan J, Wei B, Xu Z, Yang S, Zhou Y, et al (2013) Predictive value of Sox2 expression in transurethral resection specimens in patients with T1 bladder cancer. Med Oncol 30: 445. 23307254 10.1007/s12032-012-0445-z

[13] Cimpean AM, Encica S, Raica M, Ribatti D (2011) SOX2 gene expression in normal human thymus and thymoma. Clin Exp Med 11: 251–254. 21190055 10.1007/s10238-010-0127-0

[14] Gallo M, Ho J, Coutinho FJ, Vanner R, Lee L, et al (2013) A tumorigenic MLL-homeobox network in human glioblastoma stem cells. Cancer Res 73: 417–427. 23108137 10.1158/0008-5472.CAN-12-1881

[15] Hindson BJ, Ness KD, Masquelier DA, Belgrader P, Heredia NJ, et al (2011) High-throughput droplet digital PCR system for absolute quantitation of DNA copy number. Anal Chem 83: 8604–8610. 22035192 10.1021/ac202028g PMC3216358

[16] Pinheiro LB, Coleman VA, Hindson CM, Herrmann J, Hindson BJ, et al (2012) Evaluation of a droplet digital polymerase chain reaction format for DNA copy number quantification. Anal Chem 84: 1003–1011. 22122760 10.1021/ac202578x PMC3260738

[17] Benlloch S, Paya A, Alenda C, Bessa X, Andreu M, et al (2006) Detection of BRAF V600E mutation in colorectal cancer: comparison of automatic sequencing and real-time chemistry methodology. J Mol Diagn 8: 540–543. 17065421 10.2353/jmoldx.2006.060070 PMC1876165

[18] Johnson BE, Mazor T, Hong C, Barnes M, Aihara K, et al (2014) Mutational analysis reveals the origin and therapy-driven evolution of recurrent glioma. Science 343: 189–193. 24336570 10.1126/science.1239947 PMC3998672

[19] Weisenberger DJ, Siegmund KD, Campan M, Young J, Long TI, et al (2006) CpG island methylator phenotype underlies sporadic microsatellite instability and is tightly associated with BRAF mutation in colorectal cancer. Nat Genet 38: 787–793. 16804544 10.1038/ng1834

[20] Dahlin AM, Palmqvist R, Henriksson ML, Jacobsson M, Eklof V, et al (2010) The role of the CpG island methylator phenotype in colorectal cancer prognosis depends on microsatellite instability screening status. Clin Cancer Res 16: 1845–1855. 20197478 10.1158/1078-0432.CCR-09-2594

[21] Eklof V, Wikberg ML, Edin S, Dahlin AM, Jonsson BA, et al (2013) The prognostic role of KRAS, BRAF, PIK3CA and PTEN in colorectal cancer. Br J Cancer 108: 2153–2163. 23660947 10.1038/bjc.2013.212 PMC3670497

[22] Cantwell-Dorris ER, O'Leary JJ, Sheils OM (2011) BRAFV600E: implications for carcinogenesis and molecular therapy. Mol Cancer Ther 10: 385–394. 21388974 10.1158/1535-7163.MCT-10-0799

[23] Haugsten EM, Wiedlocha A, Olsnes S, Wesche J (2010) Roles of fibroblast growth factor receptors in carcinogenesis. Mol Cancer Res 8: 1439–1452. 21047773 10.1158/1541-7786.MCR-10-0168

[24] Turner N, Grose R (2010) Fibroblast growth factor signalling: from development to cancer. Nat Rev Cancer 10: 116–129. 20094046 10.1038/nrc2780

[25] Liu K, Lin B, Zhao M, Yang X, Chen M, et al (2013) The multiple roles for Sox2 in stem cell maintenance and tumorigenesis. Cell Signal 25: 1264–1271. 23416461 10.1016/j.cellsig.2013.02.013 PMC3871517

[26] Ben-Porath I, Thomson MW, Carey VJ, Ge R, Bell GW, et al (2008) An embryonic stem cell-like gene expression signature in poorly differentiated aggressive human tumors. Nat Genet 40: 499–507. 18443585 10.1038/ng.127 PMC2912221

[27] Tang XB, Shen XH, Li L, Zhang YF, Chen GQ (2013) SOX2 overexpression correlates with poor prognosis in laryngeal squamous cell carcinoma. Auris Nasus Larynx 40: 481–486. 23462687 10.1016/j.anl.2013.01.003

[28] Pham DL, Scheble V, Bareiss P, Fischer A, Beschorner C, et al (2013) SOX2 expression and prognostic significance in ovarian carcinoma. Int J Gynecol Pathol 32: 358–367. 23722508 10.1097/PGP.0b013e31826a642b

[29] Lou X, Han X, Jin C, Tian W, Yu W, et al (2013) SOX2 targets fibronectin 1 to promote cell migration and invasion in ovarian cancer: new molecular leads for therapeutic intervention. OMICS 17: 510–518. 23895273 10.1089/omi.2013.0058 PMC3783972

[30] Xiang R, Liao D, Cheng T, Zhou H, Shi Q, et al (2011) Downregulation of transcription factor SOX2 in cancer stem cells suppresses growth and metastasis of lung cancer. Br J Cancer 104: 1410–1417. 21468047 10.1038/bjc.2011.94 PMC3101944

[31] Girouard SD, Laga AC, Mihm MC, Scolyer RA, Thompson JF, et al (2012) SOX2 contributes to melanoma cell invasion. Lab Invest 92: 362–370. 22184093 10.1038/labinvest.2011.188 PMC3887365

[32] Peyssonnaux C, Eychene A (2001) The Raf/MEK/ERK pathway: new concepts of activation. Biol Cell 93: 53–62. 11730323 10.1016/s0248-4900(01)01125-x

[33] Baldus SE, Schaefer KL, Engers R, Hartleb D, Stoecklein NH, et al (2010) Prevalence and heterogeneity of KRAS, BRAF, and PIK3CA mutations in primary colorectal adenocarcinomas and their corresponding metastases. Clin Cancer Res 16: 790–799. 20103678 10.1158/1078-0432.CCR-09-2446

[34] Benvenuti S, Sartore-Bianchi A, Di Nicolantonio F, Zanon C, Moroni M, et al (2007) Oncogenic activation of the RAS/RAF signaling pathway impairs the response of metastatic colorectal cancers to anti-epidermal growth factor receptor antibody therapies. Cancer Res 67: 2643–2648. 17363584 10.1158/0008-5472.CAN-06-4158

[35] Samowitz WS, Sweeney C, Herrick J, Albertsen H, Levin TR, et al (2005) Poor survival associated with the BRAF V600E mutation in microsatellite-stable colon cancers. Cancer Res 65: 6063–6069. 16024606 10.1158/0008-5472.CAN-05-0404

[36] Ogino S, Nosho K, Kirkner GJ, Kawasaki T, Meyerhardt JA, et al (2009) CpG island methylator phenotype, microsatellite instability, BRAF mutation and clinical outcome in colon cancer. Gut 58: 90–96. 18832519 10.1136/gut.2008.155473 PMC2679586

[37] Phipps AI, Buchanan DD, Makar KW, Win AK, Baron JA, et al (2013) KRAS-mutation status in relation to colorectal cancer survival: the joint impact of correlated tumour markers. Br J Cancer 108: 1757–1764. 23511557 10.1038/bjc.2013.118 PMC3668469

[38] Jang JH (2005) Reciprocal relationship in gene expression between FGFR1 and FGFR3: implication for tumorigenesis. Oncogene 24: 945–948. 15558020 10.1038/sj.onc.1208254

[39] Sato T, Oshima T, Yoshihara K, Yamamoto N, Yamada R, et al (2009) Overexpression of the fibroblast growth factor receptor-1 gene correlates with liver metastasis in colorectal cancer. Oncol Rep 21: 211–216. 19082464

[40] Yang F, Gao Y, Geng J, Qu D, Han Q, et al (2013) Elevated expression of SOX2 and FGFR1 in correlation with poor prognosis in patients with small cell lung cancer. Int J Clin Exp Pathol 6: 2846–2854. 24294370 PMC3843264

