# Supplementary material for: SOX2 Expression Is Regulated by BRAF and Contributes to Poor Patient Prognosis in Colorectal Cancer
Source: PLoS One. 2014 Jul 10;9(7):e101957. doi: 10.1371/journal.pone.0101957 (PMC4092103; doi:10.1371/journal.pone.0101957)
Supplement: Figure S1 — Successful transfection of pMCEF-BRAFV600E or pcDNA3-KRASG12V into Caco2 colon cancer cell line. (PDF) [file pone.0101957.s001.pdf]

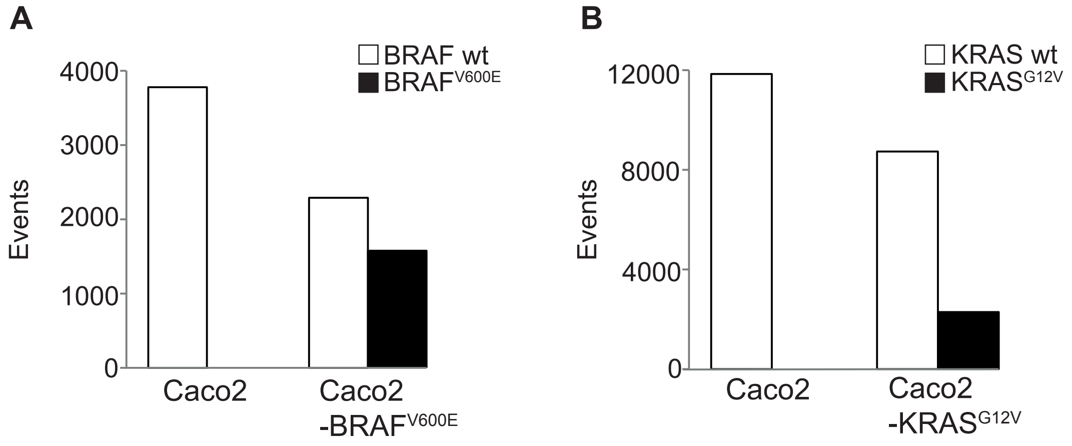

Figure S1: Successful transfection of pMCEF-BRAF^{V600E} or pcDNA3-KRAS^{G12V} into Caco2 colon cancer cell line. BRAF^{V600E} (A) or KRAS^{G12V} (B) was detected by digital droplet PCR, using the Bio-RadQX200 ddPCR platform.
