# Supplementary material for: SOX2 Expression Is Regulated by BRAF and Contributes to Poor Patient Prognosis in Colorectal Cancer
Source: PLoS One. 2014 Jul 10;9(7):e101957. doi: 10.1371/journal.pone.0101957 (PMC4092103; doi:10.1371/journal.pone.0101957)
Supplement: Figure S2 — Caco2-SOX2 cells have an increased SOX2 expression at both mRNA and protein level. (PDF) [file pone.0101957.s002.pdf]

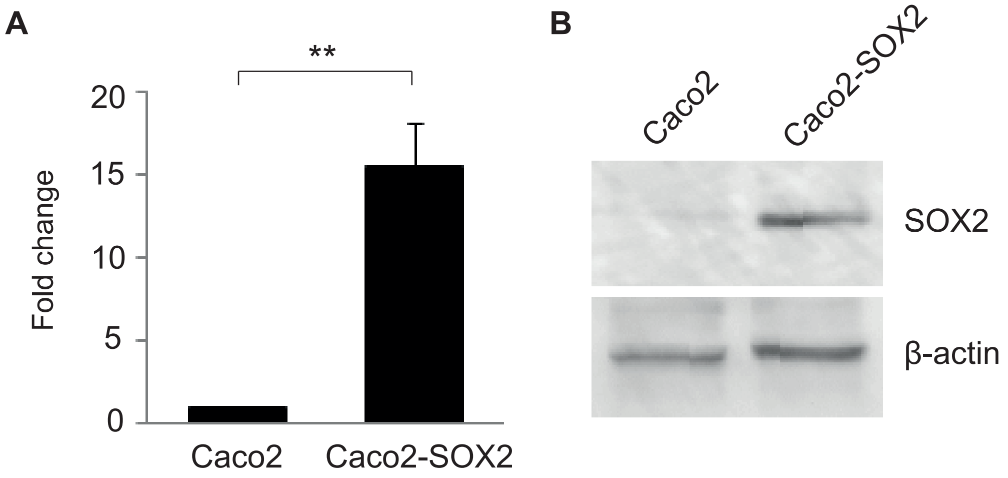

Figure S2. Caco2-SOX2 cells have an increased SOX2 expression at both mRNA and protein level. (A) SOX2 expression by RT-PCR analysis in Caco2 cells and Caco2 cells stably overexpressing SOX2 (Caco2-SOX2). (B) Western blot analysis showing that Caco2-SOX2 cells have increased protein expression of SOX2 compared to Caco2 cells. **p < 0.01.
